# Randomized, Double-Blind, Placebo-Controlled Clinical Trial Assessing the Efficacy of *Lacticaseibacillus rhamnosus* CRL 1505 in Preventing Upper Respiratory Tract Infections in Healthy Adults

**DOI:** 10.3390/microorganisms14061270

**Published:** 2026-06-04

**Authors:** Valentina Taverniti, Ines Martinez, Beatrice Tavazzani, Carlos Baeza-Martínez, Francisco López-Garcia, Carmen Carazo-Díaz, Juan Aguera Santos, Julio Villena, Susana Salva, María Pía Taranto, Susana Álvarez, Graciela Font, Vicente Navarro-López

**Affiliations:** 1 Sacco Srl, Alessandro Manzoni Street, 29/A, 22071 Cadorago, Italy; 2 Servicio de Neumología, Hospital Universitario de Elche, 03203 Alicante, Spain; 3 Servicio de Medicina Interna, Hospital Universitario de Elche, 03203 Alicante, Spain; 4 Applied Statistical Methods in Medical Research Group, Catholic University of Murcia (UCAM), 30107 Murcia, Spain; 5 Human Microbiome Research Group, Universidad Católica San Antonio de Murcia (UCAM), 30107 Murcia, Spain; vicente.navarro@bioithas.com; 6 Reference Center for Lactobacilli [CERELA-CONICET], Chacabuco 145, San Miguel de Tucumán T4000ILC, Argentina

**Keywords:** probiotics, immune system, WURSS-21, common cold, gut-lung axis

## Abstract

Upper respiratory tract infections (URTI) are highly prevalent worldwide. Although probiotics have shown potential in preventing URTI, evidence in healthy adults remains limited. *Lacticaseibacillus rhamnosus* CRL 1505 is a strain with immunomodulatory effects in preclinical studies and benefits in healthy children. Based on this evidence, a randomized, double-blind, placebo-controlled, parallel-group clinical trial was conducted in healthy adults, receiving either *L. rhamnosus* CRL 1505 (1 bln/day) or placebo for 12 weeks, with a 4-week follow-up. The primary endpoint was the proportion of participants experiencing URTI episodes (at least one, two, or three episode(s)). The secondary endpoints included: number and duration of URTI episodes, URTI-free time, symptom severity, use of symptomatic medication, salivary IgA levels, and safety outcomes. Results show that *L. rhamnosus* CRL 1505 significantly reduced the number of participants experiencing ≥3 URTI episodes at 16 weeks compared with placebo. The probiotic group experienced fewer URTI episodes per participant, a shorter cumulative duration of URTIs, and a higher URTI-free time rate. Probiotic supplementation significantly reduced the use of symptomatic medications. In conclusion, daily supplementation with *L. rhamnosus* CRL 1505 reduced the burden of URTI in healthy adults, specifically of those experiencing more episodes, by decreasing infection frequency, duration, and medication use.

## 1. Introduction

Upper respiratory tract infections (URTIs) are a category of infectious diseases primarily caused by viruses and occasionally by bacteria. URTIs in adults comprise pharyngitis, laryngitis, acute sinusitis, otitis media, tonsilitis, and the common cold, which can involve multiple anatomical sites within the upper respiratory tract, manifesting as a diverse and fluctuating symptomatology. Although self-limiting in most cases, URTI can lead to significant respiratory complications, including bacterial superinfections, progression to lower respiratory tract infections and exacerbation of asthma or chronic obstructive pulmonary disease (COPD), which are of particular concern in patients with underlying chronic respiratory diseases. These complications are more prevalent among at-risk populations, including the elderly, individuals with diabetes, obesity, immunocompromised individuals, and those with chronic respiratory diseases, representing a substantial source of morbidity [1]. Additionally, URTIs exert a worldwide impact on the economy and healthcare systems [2] by increasing healthcare demand and expenditures and causing workplace and school absenteeism, a reduction in overall productivity, and misuse of antibiotics. Considering that the incidence rate of URTIs is the highest among infectious diseases [1], the development of preventive measures to mitigate their impact should be a priority. Probiotics are live microorganisms that, when administered in adequate amounts, confer a health benefit on the host [3]. A recent Cochrane systematic review of randomized controlled trials has shown that probiotic treatment is effective in preventing URTI by reducing both the incidence and duration of each episode [4]. However, these studies exhibit considerable heterogeneity. Moreover, while the precise mechanisms of action of probiotics have not been fully elucidated, research suggests several potential pathways through which they may exert their effects, primarily by enhancing the immune response, competing and antagonizing different pathogens through multiple strategies, strengthening the gut mucosal barrier, and modulating the gut microbiota and the release of beneficial microbial metabolites [5,6,7,8]. In this context, *Lacticaseibacillus rhamnosus* CRL 1505, a probiotic strain originally isolated from goat milk, is supported by an extensive body of preclinical evidence accumulated over a decade of research and more than 50 publications, which also has several demonstrated effects for respiratory health in mice when orally administered, by boosting immune responses against diverse pathogenic microbes and inducing specific antiviral defenses, while dampening inflammation [9,10,11]. A randomized, placebo-controlled clinical trial conducted in healthy children aged 2 to 5 years old demonstrated that the consumption of yogurt fortified with *L. rhamnosus* CRL 1505 reduced the incidence of URTI, tonsilitis and gastrointestinal infections [12]. The recent report “Global Impact of Respiratory Diseases”, published by the Forum of International Respiratory Societies, emphasized that respiratory infections represent a significant global public health threat and underscored the urgent need for research aimed at developing strategies to improve the prevention of these diseases [13]. In this perspective, we evaluated whether *L. rhamnosus* CRL 1505 can prevent URTI in healthy adults, addressing a preventive approach that could have meaningful implications for respiratory health.

## 2. Materials and Methods

### 2.1. Study Design and Ethical Considerations

The present clinical trial features a randomized, double-blind, parallel-group, placebo-controlled design, with a 12-week intervention period followed by an additional 4-week follow-up period without intervention. The study was designed to coincide with the peak months of URTI incidence, with inclusion beginning in October and November 2023 and follow-up concluding between January and February 2024.

The study was conducted in accordance with the Declaration of Helsinki and its later amendments. Ethical approval was obtained from the Ethics Committee of the Universidad Católica San Antonio de Murcia, Spain (approval code CE092303), and written informed consent was obtained from all participants prior to enrolment. The study was registered in the ClinicalTrial.gov repository with the ID NCT07091955 “https://clinicaltrials.gov/study/NCT07091955” (accessed on 21 July 2025) and in the public repository of the Universidad Católica San Antonio de Murcia “https://repositorio.ucam.edu/handle/10952/7702” (accessed on 2 October 2023).

### 2.2. Recruitment and Participant Selection Criteria

Participants were recruited from the Human Microbiome Research group at the Universidad Católica San Antonio de Murcia. Eligible candidates were then invited for an in-person interview with the physicians participating in the study and received detailed explanations of the trial procedures and provided written informed consent. Those that met all the inclusion and exclusion criteria were finally included in the study.

The included population consisted of healthy men and women aged 18 to 65 years with a body mass index (BMI) lower than 35 kg/m^2^. The exclusion criteria primarily consisted of individuals with acute or chronic diseases and those with an immunocompromised health status. These clinical conditions included metabolic disorders such as diabetes or obesity (BMI greater than 35.1 kg/m^2^); congenital and acquired immune defects (including allergies); nasal ulcers or polyps causing nasal obstruction; abuse of alcohol, tobacco, or other substances; pregnancy or breastfeeding; influenza vaccination within the previous six months; or ongoing treatment with medications or dietary supplements that could influence outcomes. This included use of immunosuppressants, immune stimulants (such as echinacea supplements), and other substances like analgesics, anti-inflammatory drugs, antitussives/expectorants, anti-flu preparations, decongestants, antibiotics, antihistamines, and probiotics within the four weeks prior to the start of the study.

### 2.3. Study Groups and Interventions

Included participants were randomized into two treatment groups at a 1:1 allocation ratio. The study groups were differentiated by the type of treatment received: probiotic or placebo. The probiotic product was a lyophilized preparation containing *L. rhamnosus* CRL 1505, with a concentration of 1 × 10^9^ colony-forming units (CFUs) per daily dose. The CRL 1505 strain belongs to the culture collection of the Reference Center for Lactobacilli (CERELA-CONICET). A placebo with the same appearance as the active product was used as the comparator and contained only corn starch and maltodextrin as excipients. The daily dose for both products was one capsule per day for 12 weeks. In addition, participants were instructed not to alter their diet or physical activity during the study period.

### 2.4. Randomization and Blinding

A block randomization method was employed to ensure balance in the number of subjects assigned to each intervention group. After inclusion, participants were assigned consecutively according to a computer-generated, pre-established randomization sequence based on the aforementioned method. The treatment (probiotic or placebo) was unknown to both the researchers and the participants, as the study followed a double-blind design. The treatments were labeled with the protocol and randomization code and did not display any distinctive features indicating the treatment group.

### 2.5. Outcomes, Procedures and Data Analysis

Participants’ baseline clinical and demographic data were collected and they were required to complete an online questionnaire daily throughout the clinical trial Online Supplemental Material). Data for outcomes analysis was extracted from these responses. In addition, at baseline and at the end of the intervention period (week 12), participants were instructed to collect saliva samples using a Salivette^®^ device (SARSTEDT AG & Co. KG, Nümbrecht, Germany). The quantification of IgA levels was performed using a commercial ELISA kit (DiaMetra Srl Unipersonale, Spello, Perugia, Italy) in accordance with the manufacturer’s instructions. A saliva sample was obtained for immunoglobulin A (IgA) before starting the intervention. The complete procedure is detailed in the Online Supplemental Material.

The primary endpoint was the difference between study groups in the proportion of participants diagnosed with at least 1, 2, or 3 URTI episodes. For this purpose, it was crucial to establish precise criteria for defining a common cold episode. The onset of a common cold episode was defined by clinicians participating in the study when patients meet two criteria for at least two consecutive days: (i) responding “yes” to the question, “Do you think you have a cold or are you getting a cold?’ and (ii) achieving a score of at least two points on the Jackson scale [14] (Online Supplemental Material). The end of a common cold episode was defined as the last day of symptoms, followed by at least two consecutive symptom-free days.

As secondary endpoints, the trial evaluated the following: the difference between study groups in the number of URTI episodes per participant; the proportion of participants who developed URTI complications (bacterial infections such as pneumonia, otitis media, or acute sinusitis); the time to the first URTI episode (in days); the duration of URTI episodes per participant (days with URTI/participants); the URTI-free time rate (proportion of accumulated days without URTI symptoms relative to the total study period for all participants); the “Wisconsin Upper Respiratory Symptoms Survey 21” (WURSS-21, Online Supplemental Material) score per day of common cold [15]; the proportion of participants requiring antibiotics; the proportion of URTI episodes and proportion of days with URTI where symptomatic medication was used (including self-administration of analgesics, antipyretics, anti-inflammatories, antitussives, expectorants, decongestants, antihistamines, or anti-influenza preparations); and the proportion of participants who experienced gastrointestinal infections.

The safety profile of the study product was assessed by monitoring adverse events (AEs), including their incidence and severity, throughout the intervention period.

### 2.6. Sample Size Estimation

Using G*Power (version 3.1.9.7), the sample size was calculated assuming 50% of healthy adults would experience at least one URTI episode, with a 23% reduction expected between the probiotic and placebo groups (4). With a significance level of 0.05, a power of 0.8, and a 15% drop-out rate, 140 participants (70 per group) were required.

### 2.7. Statistical Analysis

Baseline demographic data was used to describe the study population and assess intergroup homogeneity. Descriptive quantitative variables were summarized as mean and standard deviation (SD), and categorical variables as proportions. Data was analyzed as a modified intention to treat (mITT) approach. Group comparisons for continuous variables were conducted using the *t*-test, while proportions were compared with the Chi-square test. To control potential confounders, a multiple linear regression model was applied with treatment type as the main explanatory variable and demographic and contextual variables included as covariates. No confounding variables or relevant interactions were found. Data was analyzed with R statistical software (version 4.1.3).

## 3. Results

### 3.1. Included Participants Characteristics

**Figure 1:**
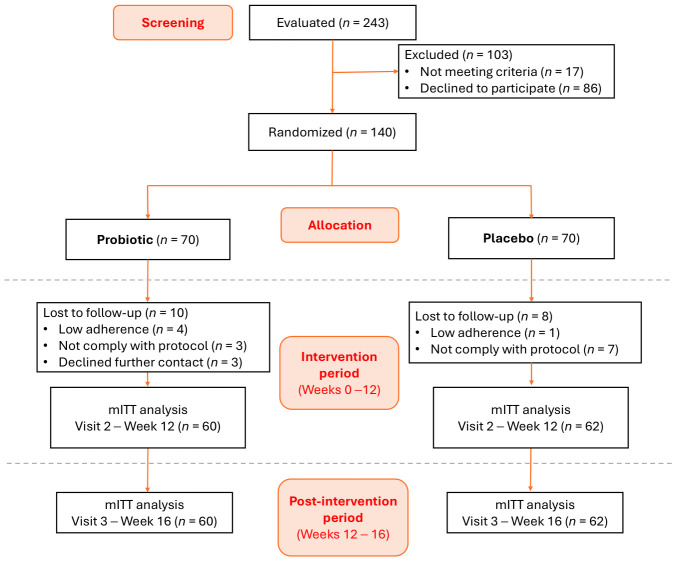
CONSORT diagram of participant flow.

A total of 140 subjects were randomized in the study (Table 1). The data from 60 subjects in the probiotic group and 62 in the placebo group who completed at least two visits during the study were included in the analysis. The remaining participants withdrew from the study or declined further contact after the baseline visit and therefore did not complete the follow-up during the intervention and post-treatment periods. Figure 1 illustrates participant screening, randomization, and the final cases included in the analysis, along with reasons for exclusion and the number of subjects lost to follow-up for each intervention group.

### 3.2. Primary Endpoint

Concerning the primary endpoint of the trial, although a smaller proportion of individuals in the probiotic group experienced at least 1 or 2 URTI episodes compared to the placebo group, these differences did not reach statistical significance. In this regard, at 16 weeks, the proportion of participants with ≥2 URTI episodes was 15.5% lower in the probiotic group compared to the placebo group, approaching statistical significance. For those patients with ≥3 URTI episodes, a trend toward statistical significance was noted, with an 8% reduction already observed after 12 weeks of intervention. After 16 weeks a significant reduction of 12.7% was observed in the probiotic group compared to the placebo group (*p* = 0.03) (Table 2).

### 3.3. Secondary Endpoints

#### 3.3.1. URTI Episodes per Participant

Subjects receiving the probiotic *L. rhamnosus* CRL 1505 experienced significantly fewer URTI episodes than the placebo group after 16 weeks (53 vs. 78 total URTI episodes with a reduction of −0.37 URTI episodes per participant; *p* = 0.05) (Table 3), with a trend in reduction appearing already at 12 weeks (46 vs. 65 total URTI episodes with a reduction of −0.28 URTI episodes per participant; *p* = 0.09).

#### 3.3.2. URTI Complications per Participant

No URTI complications (pneumonia, otitis media, or acute sinusitis) were diagnosed in either the probiotic or placebo group during the 16-week follow-up.

#### 3.3.3. Time to First URTI Episode per Participant

There were no significant differences in the days to the first URTI episode between study groups over the 12-week intervention period (probiotic: 36.8 days [SE 3.7] vs. placebo: 38.6 days [SE 3.6]; *p* = 0.72).

#### 3.3.4. Duration of URTI Episodes per Participant

For subjects treated with *L. rhamnosus* CRL 1505, the mean number of days with URTI per participant was significantly lower at 16 weeks compared to the placebo group (*p* = 0.03), showing a mean reduction of approximately 3 days (Table 4). After 12 weeks of intervention, the difference trended towards statistical significance (*p* = 0.07), with a mean reduction of 2.34 days.

#### 3.3.5. URTI-Free Time Rate

Participants treated with *L. rhamnosus* CRL 1505 exhibited a 2.9% higher URTI-free time rate compared to those who received placebo, with this difference being statistically significant (Table 5).

#### 3.3.6. WURSS-21 Score by Day of Common Cold

The overall severity of each day of common cold episodes, as assessed by the WURSS-21 questionnaire score, was similar between subjects treated with *L. rhamnosus* CRL 1505 and those receiving placebo, with no significant differences at 12 or 16 weeks. The complete data for this comparison are provided in the Online Supplemental Material.

#### 3.3.7. Proportion of Participants Requiring Antibiotic Treatment

Throughout the 16-week follow-up period, antibiotic treatment prescribed by a physician was required for only one URTI episode in both the probiotic and placebo groups. Due to the limited number of cases, no further analysis was conducted.

#### 3.3.8. Proportion of URTI Episodes and Proportion of Days with URTI Where Symptomatic Medication Was Used

Most of the symptomatic medication was self-reported by participants and subsequently reviewed and validated by the study physician at 12 and 16 weeks, with only three URTI episodes in the placebo group requiring a medical prescription. Importantly, the analysis excluded antibiotic medication, which was already accounted for.

Participants treated with *L. rhamnosus* CRL 1505 used symptomatic medication for a smaller proportion of URTI episodes and had a lower percentage of URTI days with symptomatic medication compared to the placebo group, with both differences being statistically significant (*p* = 0.001) (Table 6).

#### 3.3.9. Proportion of Participants Suffering Gastrointestinal Infections

During the 16-week follow-up, two cases of gastrointestinal infection (diarrhea) were reported in the placebo group, precluding further analysis.

#### 3.3.10. Salivary Immunoglobulin A (IgA)

After 12 weeks, salivary IgA levels decreased from baseline in both the probiotic and placebo groups, with no statistically significant difference between these changes (probiotic–placebo difference: CI 95% = −10 µg/mL [−36 to 16 µg/mL]; *p* = 0.47).

Secondary endpoints were pre-specified in the study protocol and statistical analysis plan. However, no formal adjustment for multiplicity was applied; therefore, results from secondary endpoint analyses should be interpreted with caution in the context of multiple comparisons.

### 3.4. Safety Analysis

Adverse events (AEs) were summarized descriptively and no formal statistical comparisons between groups were performed. All reported adverse events were classified as mild. A total of 18 AEs were attributed to the study product (possible relationship): 8 in the probiotic group and 10 in the placebo group, all involving the digestive system. No participants were withdrawn from the study due to AEs and none dropped out due to intolerance to the study product. Additionally, adherence was assessed calculating the proportion of returned study product units relative to the total number of units dispensed, adjusted for the duration of treatment. Among the 122 participants who returned the treatment for counting, the adherence exceeded 95% in both groups, indicating a high rate of compliance.

The full data on the number and types of AEs during the 12-week intervention in both study groups are provided in the Online Supplemental Material.

## 4. Discussion

The findings of this clinical trial support the beneficial effects of *L. rhamnosus* CRL 1505 in reducing the burden of URTI in adults, in particular for recurrent episodes. Despite numerous published trials evaluating the efficacy of probiotics on the incidence, duration, and severity of respiratory infections in children [16,17,18], evidence specifically addressing their impact on URTI in healthy adults remains limited. Even though probiotic interventions showed encouraging results on the prevention/treatment of URTI in adults, overall clinical trials exhibit heterogeneity in their design, methodology, and interventions, posing a challenge for the comparative analysis of outcomes [4,19,20]. Despite this, some conclusions have been proposed for the use of probiotics for URTI, in particular that probiotics may be beneficial in preventing at least one occurrence of URTI and are likely beneficial in preventing at least three occurrences of URTI [4]. The results of this study are in agreement with those conclusions. *L. rhamnosus* CRL 1505 intake reduced the number of individuals contracting ≥3 URTI episodes, underscoring its potential for individuals prone to recurrent infections. Our results suggest that *L. rhamnosus* CRL 1505 intervention might be more effective in preventing infective episodes in more susceptible individuals, i.e., those with recurrent episodes, but this hypothesis needs to be further confirmed on a larger population. Recurrent respiratory tract infections (RRTIs) have been mainly defined and described in children, older people, pregnant women, people with chronic medical conditions and immunocompromised individuals [21,22,23]. RRTI can be driven by subject susceptibility (e.g., non-fully mature immune system, comorbidities, immunosenescence, etc. …) but also lifestyle and environmental factors (e.g., exposure to pollutants, housing conditions, frequent contact with young children, chronic stress, smoking, spread of antimicrobial resistance, etc.) [24,25,26]. All these aspects make the understanding and prevention of RRTI particularly important [27]. Metanalysis studies have reported that probiotics may not only reduce the incidence of URTI episodes but also their duration, compared with placebo [28]. Indeed, another benefit of *L. rhamnosus* CRL 1505 intake was the significant decrease in the number of days with URTI per participant and increase in URTI-free time. Precedent studies provide evidence of a reduction of approximately 1 day in the duration of URTI episodes [4] or respiratory infections in general [19]. For example, the 12-week administration of *L. rhamnosus* GG and *Bifidobacterium animalis* subsp. *lactis* BB12 in young adults reduced the median of URTI duration by 2 days [29]. In another study, the number of undergraduate students that reported ≥1 day of cold/flu was significantly lower than placebo during a 6-week intervention period with *B. bifidum* R0071 (−13%), but not with *Lactobacillus helveticus* R0052 or *Bifidobacterium longum* ssp. *infantis* R0033 [30].

There are limited systematic reviews that address symptom severity in URTI [19], but the study by Smith and colleagues (2003) showed that the median severity score was 34% lower in the probiotic group compared to placebo [29]. Although *L. rhamnosus* CRL 1505 did not alleviate the severity of each common cold episode, as measured by the WURSS-21 [15], importantly *L. rhamnosus* CRL 1505 intake was associated with a lower need for over-the-counter drugs. This result is of specific relevance in view of the increasing misuse of over-the-counter drugs and related safety risks [31,32]. To explore potential therapeutic mechanisms, the study also quantified salivary IgA. No significant modulation was observed with *L. rhamnosus* CRL 1505 treatment, unlike the clinical trial conducted in children [12]. However, the trial in children used a 6-month intervention and a different ELISA method for salivary IgA quantification and employed a distinct sample collection method. In addition, although biologically plausible, the current evidence indicates that salivary IgA is not yet a reliable URTI biomarker due to problems of reproducibility, high interindividual variability and confounding factors [33].

Because no other immune marker was assessed, we can only propose mechanistic hypotheses for the beneficial effects of *L. rhamnosus* CRL 1505 observed in this study. Diverse murine models have clearly shown the capacity of *L. rhamnosus* CRL 1505 to modulate the immune system [9,10,11], in particular its anti-inflammatory capacity in the context of viral infections through IL-10-mediated mechanisms [9,34]. In addition, increased levels of circulating plasmacytoid dendritic cells and higher expression of antiviral markers, such as interferon-α and MX dynamic-like GTPase, were observed when heat-inactivated cells of *L. rhamnosus* CRL 1505 were administered to healthy adults [35,36]. However, the activations of this type of response remain speculative for this trial. A limitation of the present study is its reliance on a clinical diagnosis based on the use of the Jackson scale [14], as it has several limitations that hinder its universal applicability for diagnosing the common cold [37]. To address this, symptom data reported directly by patients were used, with the final diagnosis reviewed and validated by a clinician. In addition, no virological confirmation of URTI episodes was performed; therefore, the viral etiology of each episode and the specific respiratory pathogens involved could not be confirmed. Nevertheless, the term “common cold” is widely used in the medical literature, although its precise definition remains challenging and partly subjective. A diagnostic strategy aimed at assigning URTI to specific anatomical sites is clinically difficult, due to the considerable overlap and temporal variability of symptoms. This variability is partly explained by the fact that more than 200 recognized viruses can cause URTI, and the clinical presentation is largely influenced by factors such as age, immune system status, and the presence of comorbidities [38]. Importantly, virological testing is not routinely required for the diagnosis of uncomplicated common cold episodes in clinical practice, which is generally based on typical upper respiratory tract symptoms. In this context, the Jackson scale provides a structured and standardized method to capture symptoms commonly associated with the common cold. Thus, although the absence of virological confirmation limits pathogen-specific or mechanistic interpretations, the symptom-based definition with clinician confirmation used in this study remains clinically relevant. Accordingly, the findings should be interpreted as referring to self-reported, symptom-defined URTI-like episodes rather than virologically confirmed infections. Despite efforts to mitigate the inherent limitations of this disease construct, the results reported in this study need to be further explored.

## 5. Conclusions

*L. rhamnosus* CRL 1505 demonstrated beneficial effects in a healthy adult population, alleviating URTI burden, in particular the reduction in recurrent episodes. Considering that URTI often act as triggers for exacerbations of chronic respiratory diseases, these findings could be valuable for guiding future research. The limited number of studies focusing on probiotic use to alleviate URTI in the adult population make this study especially relevant in trying to address the existing gap in knowledge.

## Figures and Tables

**Table 1:** Baseline demographic and clinical data.

	Probiotic (*n* = 70)	Placebo (*n* = 70)
Sex (♂), *n* (%)	47 (67.1%)	55 (78.6%)
Age (years), mean ± SD	28.0 ± 10.7	28.0 ± 10.4
BMI (kg/m^2^), mean ± SD	23.2 ± 3.3	22.7 ± 3.2
Professional activity:		
-Student, *n* (%)	46 (65.7%)	43 (61.4%)
-Health worker, *n* (%)	2 (2.8%)	6 (8.6%)
-Professor, *n* (%)	6 (8.6%)	6 (8.6%)
-Other, *n* (%)	16 (22.8%)	15 (21.4%)
Concurrent disease ^A^, *n* (%)	4 (5.7%)	5 (7.1%)
-Neurological, *n*	1	0
-Dermatological, *n*	1	1
-Endocrinological, *n*	0	1
-Psychiatric, *n*	0	1
-Cardiovascular, *n*	1	1
-Other, *n*	1	1
Continuous pharmacological treatment ^B^, *n* (%)	0 (0%)	3 (4.8%)
-Antidepressants, *n*	0	1
-Oral contraceptives, *n*	0	1
-Antihypertensives, *n*	0	1
Continuous contact with individuals at risk for URTI (elderly, children, immunocompromised), *n* (%)	14 (20.0%)	19 (27.1%)
URTI episodes during the 3 months prior to the beginning of the study, *n* (%)	21 (30.0%)	27 (38.6%)
-Common cold, *n*	21	27
-Influenza, *n*	0	0
-Bacterial infection, *n*	0	1
URTI episodes during the 3 months prior to the beginning of the study, mean ± SD	0.3 ± 0.5	0.4 ± 0.5
Salivary IgA (µg/mL), mean ± SD	60 ± 70	54 ± 62

^A^ Concurrent clinical condition presented by these participants [always of mild severity], which the researcher deemed would not influence the efficacy results of the study product, was not considered an exclusion criterion in these cases. ^B^ Continuous or habitual medication that the researcher deemed would not influence the efficacy results of the study product was not considered an exclusion criterion in these cases.

**Table 2:** Proportion of subjects who presented URTI.

URTI Episodes	Period	Probiotic Group ^A^	Placebo Group ^A^	Difference ^B^	*p*-Value ^C^
≥1	12 weeks	33 (55.0%)	40 (64.5%)	−9.5% (−26.8% to 7.8%)	0.28
	16 weeks	37 (61.7%)	42 (67.7%)	−6.0% (−23.0% to 10.9%)	0.48
≥2	12 weeks	12 (20.0%)	19 (30.6%)	−10.6% (−25.9% to 4.7%)	0.18
	16 weeks	13 (21.6%)	23 (37.1%)	−15.5% (−31.3% to 0.5%)	0.06
≥3	12 weeks	1 (1.7%)	6 (9.7%)	−8% (−16.1% to 0.0%)	0.06
	16 weeks	3 (5.0%)	11 (17.7%)	−12.7% (−23.7% to −1.7%)	0.03 *

^A^ N (%). ^B^ 95%CI treatment effect (probiotic–placebo). ^C^ Chi-square test (* statistically significant difference).

**Table 3:** URTI episodes per participant.

Period	Probiotic Group ^A^	Placebo Group ^A^	Difference ^B^	*p*-Value ^C^
12 weeks	0.77 (0.11)	1.05 (0.13)	−0.28 (−0.61 to 0.04)	0.09
16 weeks	0.88 (0.11)	1.26 (0.15)	−0.37 (−0.74 to −0.01)	0.05 *

^A^ Mean (Standard Error). ^B^ Mean (95%CI). ^C^ *T*-test (* statistically significant difference).

**Table 4:** Days with URTI episode per participant.

Period	Probiotic Group ^A^	Placebo Group ^A^	Difference ^B^	*p*-Value ^C^
12 weeks	4.35 (0.81)	6.69 (0.99)	−2.34 (−4.90 to 0.21)	0.07
16 weeks	4.77 (0.81)	7.77 (1.10)	−3.01 (−5.73 to −0.29)	0.03 *

^A^ Mean (Standard Error). ^B^ Mean (95%CI). ^C^ *T*-test (* statistically significant difference).

**Table 5:** URTI-free time rate [%].

Period	Probiotic Group	Placebo Group	Difference ^A^	*p*-Value ^B^
12 weeks	94.8%	91.9%	2.9% (1.9% to 3.9%)	<0.0001 *
16 weeks	96.0%	93.1%	2.9% (2.2% to 3.7%)	<0.0001 *

^A^ 95%CI treatment effect (probiotic–placebo). ^B^ Chi-square test (* statistically significant difference).

**Table 6:** Proportion of URTI episodes and proportion of days where symptomatic medication was used.

	Period	Probiotic Group	Placebo Group	Difference ^C^	*p*-Value ^D^
URTI Episodes where participants received symptomatic medication	12 weeks	50.0% (23/46) ^A^	64.6% (42/65) ^A^	−14.6% (−33.1% to 3.9%)	0.12
	16 weeks	49.0% (26/53) ^A^	68.0% (53/78) ^A^	−19.0% (−35.4% to −1.9%)	0.03 *
Days with URTI where participants received symptomatic medication	12 weeks	30.3% (79/261) ^B^	42.2% (175/415) ^B^	−11.9% (−19.2% to −4.6%)	0.001 *
	16 weeks	30.8% (88/286) ^B^	43.6% (210/482) ^B^	−12.8% (−19.7% to −5.9%)	0.001 *

^A^ % (Number of episodes relative to the total URTI episodes); ^B^ % (number of days relative to the total days with URTI); ^C^ 95%CI treatment effect (probiotic–placebo); ^D^ Chi-square test (* statistically significant difference).

## Data Availability

The raw data collected during the current study are available from the corresponding author upon request.

## Supplementary Materials

The following supporting information can be downloaded at https://www.mdpi.com/article/10.3390/microorganisms14061270/s1.

## Abbreviations

The following abbreviations are used in this manuscript:

CFUs	Colony-forming units
IgA	Immunoglobulin A
URTI	Upper respiratory tract infections
mITT	Modified intention to treat
