# Supplementary material for: Randomized, Double-Blind, Placebo-Controlled Clinical Trial Assessing the Efficacy of Lacticaseibacillus rhamnosus CRL 1505 in Preventing Upper Respiratory Tract Infections in Healthy Adults
Source: Microorganisms. 2026 Jun 4;14(6):1270. doi: 10.3390/microorganisms14061270 (PMC13303622; doi:10.3390/microorganisms14061270)
Supplement: Supplementary file 1 [file microorganisms-14-01270-s001.zip › microorganisms-4336491-supplementary.pdf]

SUPPLEMENTARY MATERIAL

1. Online Questionnaire.

Participants were instructed to complete an online questionnaire daily throughout the 16-week duration of the clinical trial.

Throughout the duration of the trial, the completion of the online diary was closely monitored. Participants who deviated from the correct completion protocol were promptly contacted by telephone to provide guidance and ensure adherence to the study requirements.

Each day, participants were required to answer 3 questions:

1. Do you think you have a cold or are you getting a cold? Based on the response, other follow-up questions were generated.

- If the answer was "YES", the participant was required to complete the Jackson scale:
    - If the Jackson score was also ≥2, the participant was required to complete the WURSS-21 questionnaire and to answer the following 2 questions:
        - Have you required medical attention due to the severity or complications of your cold? (If the answer was "YES", the diagnosis must be specified).
        - Are you taking any medication for the cold? (If the answer was "YES", medication must be specified).

2. Have you taken any medication today not related to upper respiratory tract infections? (If the answer was "YES", medication must be specified).

3. Do you have any other disease, or have you developed any symptoms not related to upper respiratory tract infections (If the answer was "YES", the diagnosis must be specified).

1.1 Jackson Scale.

In this scale each, typical URTI symptom is scored from 0 to 3 based on the last 24 hours: 0 (symptom absent), 1 (symptom present but not bothersome or irritating), 2 (symptom somewhat bothersome and irritating) and 3 (symptom bothersome and irritating most of the time). The 8 symptoms evaluated were sore throat, nasal congestion, nasal discharge, cough, sneezing, headache, muscle pain and chills.

1.2 WURSS-21.

The WURSS-21 consists of 21 questions that address various aspects of URTI, focusing on both the severity of symptoms and their impact on quality of life. Each item is rated on a scale from 0 to 7. The questions are:

- How sick do you feel today? (0=Not sick, 1=Very Mildly, 3=Mildly, 5=Moderately, 7=Severely).
- Please rate the average severity of your cold symptoms over the last 24 hours for each symptom (0=Do not have this symptom, 1=Very Mild, 3=Mild, 5=Moderate, 7=Severe).
    - Runny nose.
    - Plugged nose.
    - Sneezing.
    - Sore throat.
    - Scratchy throat.
    - Cough.
    - Hoarseness.
    - Head congestion.
    - Chest congestion.
    - Feeling tired.

- Over the last 24 hours, how much has your cold interfered with your ability to (0=Not at all, 1=Very Mildly, 3=Mildly, 5=Moderately, 7=Severely):
    - Think clearly.
    - Sleep well.
    - Breathe easily.
    - Walk, climb stairs, exercise.
    - Accomplish daily activities.
    - Work outside the home.
    - Work inside the home.
    - Interact with others.
    - Live your personal life.

2. WURSS-21 score by day of URTI episode during study.

Period	PROBIOTIC Group[A]	PLACEBO Group[A]	Difference[B]	P-value[C]
12 weeks	28.60(3.48)	29.13(3.48)	-0.52(-9.94 to 8.89)	0.92
16 weeks	29.52(3.05)	28.18(3.02)	1.34(-7.83 to 10.50)	0.77

[A] Mean (Standard Error).

[B] Mean (95%CI).

[C] T-test (*Statistically Significant Difference).

3. Adverse Events.

Adverse Event	PROBIOTIC Group *(N = 70)*	PLACEBO Group *(N=70)*
Digestive System *(Acidity, Dyspepsia, Diarrhea, Abdominal Discomfort, Nausea, Vomiting, Gastroenteritis, Constipation)*	9	13
Genitourinary System *(Menstrual Pain)*	2	2
Neurological System *(Headache)*	2	11
Musculoskeletal System *(Dorsalgia)*	-	2
Skin *(Palpebral Eczema)*	1	-
General discomfort and fever	1	2
Eyes *(Conjunctivitis)*	-	1
Mouth *(Dental Infection, Oral Ulcers, Herpes)*	-	5
Ear *(Otitis)*	-	1
**TOTAL**	**17**	**37**

4. Procedure for saliva sample collection and salivary IgA analysis

Saliva samples were collected using Salivette® devices (SARSTEDT AG & Co. KG, Nümbrecht, Germany). Participants were instructed not to eat, drink, chew gum, brush their teeth, or use mouthwash for at least 30 minutes before sample collection. When possible, samples were collected at a similar time of day for each participant to minimize the potential influence of circadian variation on salivary IgA levels.

For sample collection, the cotton swab from the Salivette® device was placed in the participant's mouth and kept under the tongue until sufficiently saturated with saliva. The swab was then returned to the Salivette® tube, which was immediately closed and labelled with the participant identification code and visit/time point.

After collection, samples were centrifuged at 1000 × g for 2 min to recover saliva from the swab. After centrifugation, the volume of recovered saliva was visually checked to ensure that at least 1 mL of saliva had been obtained. The clarified saliva was stored at −20 °C until analysis. Salivary IgA levels were quantified using a commercial IgA saliva ELISA kit (DiaMetra Srl Unipersonale, Spello, Perugia, Italy), an immunoenzymatic colorimetric assay for the quantitative determination of IgA in saliva, according to the manufacturer’s instructions.
